# Monetary incentives for provision of syphilis screening, Yunnan, China

**DOI:** 10.2471/BLT.17.191635

**Published:** 2017-07-01

**Authors:** Wanyue Zhang, Hongbin Luo, Yanling Ma, Yan Guo, Qingyan Fang, Zhifang Yang, Xiujie Zhang, Xiaobin Zhang, Manhong Jia, Xiang-Sheng Chen

**Affiliations:** a Yunnan Provincial Centre for Disease Control and Prevention, 158 Dongsi Road, Kunmin, China.; b National Center for Sexually Transmitted Disease Control, China Centre for Diseases Control and Prevention, Nanjing, China.

## Abstract

**Problem:**

Early detection of syphilis-infected people followed by effective treatment is essential for syphilis prevention and control.

**Approach:**

Starting in 2010 the local health authority in Yunnan province, China, developed a network of 670 service sites for syphilis testing, diagnosis and treatment or for testing-only with referral for further diagnosis and treatment. Point-of-care tests for syphilis and syphilis interventions were integrated into the existing human immunodeficiency virus (HIV) prevention and control programme. To improve the syphilis services, a pay-for-performance scheme was introduced in which providers were paid for testing and treating patients.

**Local setting:**

Yunnan province is the region hardest hit by HIV infection and disproportionately burdened with syphilis cases in China.

**Relevant changes:**

The proportion of attendees at voluntary counselling and testing clinics who were tested for syphilis increased from 46.2% (32 877/71 162) in 2010 to 98.2% (68 012/69 259) in 2015. Syphilis-infected cases treated with the recommended therapy increased from 26.6% (264/993) in 2010 to 82.5% (453/549) in 2015 at designated testing, diagnosis and treatment sites.

**Lessons learnt:**

The strategy greatly increased the uptake of syphilis testing and treatment among people at risk. Introduction of point-of-care tests for syphilis increased coverage of the testing services. Introduction of a pay-for-performance scheme seemed to motivate health-care providers to undertake syphilis intervention services.

## Résumé

**Problème:**

Le dépistage précoce de la syphilis, suivi par un traitement efficace des personnes atteintes, est essentiel pour prévenir et contrôler cette maladie.

**Approche:**

À partir de 2010, l'administration sanitaire locale de la province du Yunnan, en Chine, a développé un réseau de 670 centres de services pour le dépistage, le diagnostic et le traitement de la syphilis ou pour le dépistage uniquement, le diagnostic et le traitement étant alors effectués dans un autre établissement. Des tests de dépistage de la syphilis sur les lieux des soins et des interventions ciblant la syphilis ont été intégrés au programme existant de prévention et de contrôle du virus de l'immunodéficience humaine (VIH). Afin d'améliorer les services liés à la syphilis, un système de rémunération aux résultats a été mis en place; les prestataires étaient donc rémunérés pour procéder au dépistage et soigner les patients.

**Environnement local:**

La province du Yunnan est la région de Chine la plus durement touchée par l'infection à VIH et est confrontée à un nombre disproportionné de cas de syphilis.

**Changements significatifs:**

La proportion des personnes qui se sont rendues dans des centres de consultation et de dépistage volontaire et qui ont bénéficié d'un dépistage de la syphilis est passée de 46,2% (32 877/71 162) en 2010 à 98,2% (68 012/69 259) en 2015. Le nombre de personnes infectées par la syphilis qui ont reçu le traitement recommandé est passé de 26,6% (264/993) en 2010 à 82,5% (453/549) en 2015 dans les centres de dépistage, de diagnostic et de soins préalablement mentionnés.

**Leçons tirées:**

La stratégie a permis d'accroître considérablement le recours au dépistage et au traitement de la syphilis par les personnes vulnérables. L'utilisation de tests de dépistage de la syphilis sur les lieux des soins a permis d'augmenter le nombre de bénéficiaires des services de dépistage. La mise en place d'un système de rémunération aux résultats a semblé inciter les prestataires de soins de santé à pratiquer des interventions ciblant la syphilis.

## Resumen

**Situación:**

La detección temprana de las personas infectadas por la sífilis seguida de un tratamiento efectivo es esencial para la prevención y el control de la enfermedad.

**Enfoque:**

Desde 2010, la autoridad sanitaria local de la provincia de Yunnan, China, desarrolló una red de 670 puntos de servicio para llevar a cabo las pruebas, el diagnóstico y el tratamiento de la sífilis o solo para llevar a cabo las pruebas con derivación a diagnóstico y tratamiento posteriores. Las pruebas en el punto de atención para la sífilis y sus intervenciones se integraron en el programa de prevención y control del virus de la inmunodeficiencia humana (VIH) existente. Para mejorar los servicios relativos a la sífilis, se introdujo un programa de pago por desempeño en el que los profesionales recibían un pago por hacer pruebas y tratar a pacientes.

**Marco regional:**

La provincia de Yunnan es la región más afectada por la infección por VIH y una cantidad desproporcionada de casos de sífilis en China.

**Cambios importantes:**

La proporción de atendidos en clínicas de pruebas y asesoramiento voluntario a los que se les realizaron pruebas de la sífilis aumentó del 46,2% (32 877/71 162) en 2010 al 98,2% (68 012/69 259) en 2015. Los casos de infección por la sífilis tratados con la terapia recomendada aumentaron del 26,6% (264/993) en 2010 al 82,5% (453/549) en 2015 en los puntos de pruebas, diagnóstico y tratamiento designados.

**Lecciones aprendidas:**

La estrategia aumentó de forma considerable la aceptación de las pruebas y el tratamiento de la sífilis entre las personas en riesgo. La introducción de las pruebas de la sífilis en el punto de atención aumentó la cobertura de los servicios de pruebas. La introducción de un programa de pago por desempeño pareció motivar a los profesionales sanitarios a realizar servicios de intervención para la sífilis.

## ملخص

المشكلة يُعد الكشف المبكر عن المصابين بالزهري متبوعًا بالعلاج الفعال أمرًا أساسيًا للوقاية من الزهري والتحكم فيه.

الأسلوب بدءًا من سنة 2010، قامت الجهات المحلية المسؤولة عن الصحة بإقليم يونان في الصين بإقامة شبكة مكونة من 670 موقعًا من مواقع الخدمة لفحص الزهري، وتشخيصه، وعلاجه أو للفحص فقط مع الإحالة للعلاج لمزيد من التشخيص والعلاج. تم دمج نقط الرعاية السريرية لمرض الزهري والتدخلات المتعلقة بالزهري في برنامج الوقاية من فيروس عوز المناعة البشري (HIV) والتحكم فيه. ومن أجل تحسين خدمات الزهري، تم تقديم نظام "الأجر حسب الأداء" والذي يحصل فيه مقدمو الخدمات على أجر حسب فحص المرضى وعلاجهم.

المواقع ****المحلية يمثل إقليم يونان أكبر منطقة مصابة بعدوى فيروس عوز المناعة البشري كما أنه مثقل بشكلٍ غير متناسب بحالات الزهري في الصين.

التغيرات ****ذات ****الصلة ارتفعت نسبة المتقدمين للاستشارة وعيادات الفحص التطوعية ممن خضعوا للفحص لمرض الزهري من 46.2‏% (32877/71162) سنة 2010 إلى 98.2%‏ (68012/69259) سنة 2015. وارتفعت نسبة الحالات المصابة بالزهري الخاضعة للعلاج الموصى به من 26.6%‏ (264/993) سنة 2010 إلى 82.5‏% سنة 2015 في مواقع الفحص والتشخيص والعلاج المحددة.

الدروس ****المستفادة ساهمت الاستراتيجية في زيادة استيعاب فحص الزهري وعلاجه بين الأشخاص المعرضين للخطر. كما ساهم تقديم فحوصات الزهري في مناطق الرعاية السريرية في زيادة تغطية خدمات الفحص. ويبدو أن نظام ربط الأجر الأداء يحفز مقدمي الرعاية الصحية على تولي تقديم خدمات التدخل الطبي لمرض الزهري.

## 摘要

**问题:**

早期发现感染梅毒人员并随后开展有效治疗,对梅毒的预防和控制至关重要。

**方法:**

中国云南省的地方卫生局从 2010 年开始,创建了由 670 个服务站点构成的网络,用于开展梅毒检测、诊断和治疗,或者仅开展检测(需转诊接受进一步诊断和治疗时)。 护理现场梅毒检测和梅毒干预整合到了现有的人类免疫缺陷病毒 (HIV) 预防和控制计划中。 为改进梅毒护理服务,引入了一项绩效薪酬计划,该计划中,按照护理提供人员为患者开展的检测和治疗,向其支付薪酬。

**当地状况:**

云南省是 HIV 感染最严重的地区,梅毒感染病例在中国也远超平均水平。

**相关变化:**

前往志愿性咨询和检测诊所接受梅毒检测的人员的比例从 2010 年的 46.2% (32 877/71 162) 上升到了 2015 年的 98.2% (68 012/69 259) 。在指定检测、诊断和治疗地点接受推荐治疗的梅毒感染病例从 2010 年的 26.6% (264/993) 上升到了 2015 年的 82.5% (453/549)。

**经验教训:**

这一策略大大增加了危险人群中梅毒检测和治疗的接受率。 引入护理现场梅毒检测扩大了检测服务的覆盖范围。 引入绩效薪酬计划似乎能够激励医疗护理提供人员开展梅毒干预服务。****

## Резюме

**Проблема:**

Раннее выявление лиц, инфицированных сифилисом, и их последующее эффективное лечение необходимы для профилактики и контроля сифилиса.

**Подход:**

Начиная с 2010 года местный орган здравоохранения в провинции Юньнань, Китай, создал сеть из 670 медицинских учреждений для тестирования, диагностики и лечения сифилиса или только для тестирования с направлением на дальнейшую диагностику и лечение. Тестирование для выявления сифилиса и лечение сифилиса в медицинских учреждениях были включены в существующую программу профилактики и контроля вируса иммунодефицита человека (ВИЧ). Для повышения качества медицинских услуг при сифилисе была введена схема оплаты в зависимости от качества работы, при которой медицинским работникам оплачивалось проведение тестирования и лечения пациентов.

**Местные условия:**

Провинция Юньнань — это регион, наиболее пострадавший от ВИЧ-инфекции и несоразмерно обремененный случаями сифилиса в Китае.

**Осуществленные перемены:**

Доля посетителей консультативных клиник и аналитических лабораторий, прошедших тестирование на сифилис, увеличилась с 46,2% (32 877/71 162) в 2010 году до 98,2% (68 012/69 259) в 2015 году. Количество инфицированных сифилисом пациентов, получивших рекомендованную терапию в специализированных медицинских учреждениях для тестирования, диагностики и лечения, увеличилось с 26,6% (264/993) в 2010 году до 82,5% (453/549) в 2015 году.

**Выводы:**

Стратегия значительно повысила эффективность тестирования и лечения сифилиса среди людей, находящихся в группе риска. Внедрение тестов на сифилис в медицинских учреждениях увеличило охват проведения тестирования. Внедрение схемы оплаты в зависимости от качества работы, по-видимому, мотивировало медицинских работников к проведению медицинского вмешательства при сифилисе.

## Introduction

Syphilis is a sexually transmitted disease caused by the bacterium *Treponema pallidum* subspecies pallidum. Approximately 3 million people aged 15‒49 years were infected with syphilis in China in 2011,^1^ accounting for a large proportion of the estimated 17 721 000 cases globally in 2012.^2^ Despite continuous efforts to promote condom use,^3^ syphilis transmission has not been prevented effectively, resulting in an increase of reported cases in the country since 2000.^4^ To control the epidemic, in 2010 the health ministry in China launched its National Programme for Prevention and Control of Syphilis (2010‒2020).^5,6^

Based on evidence from pilot studies, syphilis control efforts in China include early detection of infection through enhanced screening efforts followed by treatment of infected cases. The core intervention strategy has been described as the three-by-one approach, meaning that there are three entry points to screening linked to one standardized mode of care.^5^^,^^7^ The three settings are: (i) sexually transmitted disease clinics; (ii) human immunodeficiency virus (HIV) testing clinics (usually voluntary counselling and testing centres, and methadone maintenance treatment centres); and (iii) out-of-clinic settings where outreach services are delivered to sex workers and men who have sex with men. Standardized care provided to infected people consists of treatment with benzathine benzylpenicillin, behavioural interventions and partner notification.^5^ Screening also started in antenatal clinics after 2011 when syphilis was integrated into a separate programme for prevention of mother-to-child transmission of HIV and hepatitis B virus.^8^ We describe the approaches used to scale-up the syphilis screening and treatment interventions in Yunnan province.

## Local setting

Yunnan province, with a population of 47 million in 2015, is located in the south-west of the country and is the region hardest hit by HIV infection in China. By the end of 2010, the cumulative reported number of people with HIV infection and acquired immune deficiency syndrome (AIDS) in Yunnan was 83 925; accounting for 21.0% of the total national figure of 399 643.^9^ The province is also disproportionately burdened with syphilis cases. Approximately 7000 people with different stages of syphilis infection were reported in 2011 among 45 966 362 residents, an incidence of 15 per 100 000 people.^10^ To respond effectively to the increasing epidemic of HIV, a province-wide campaign to prevent and control HIV/AIDS was initiated in Yunnan in 2005. For the initiative, the Yunnan government established a functional network of HIV testing services in health facilities, which provided opportunities for integrating interventions targeted at other sexually transmitted diseases. In addition, introduction of point-of-care tests for syphilis in China allowed an expansion of syphilis testing in clinics and out-of-clinic settings, such as outreach services or sometimes pharmacies, in the province.^11^ Moreover, the central government issued a specific policy to ensure the availability of benzathine benzylpenicillin in health facilities for syphilis treatment.^7^ Supplies were ensured through advocacy efforts together with site supervision and monitoring.

## Approach

After the three-by-one approach was proposed as the core technical strategy, the next step was to implement the strategy in the field. To do this, the Yunnan Provincial Department of Health allocated public health funds for syphilis testing and treatment services.

Through a situation analysis, the local health facilities were mapped, evaluated for capacity and then categorized as sites for syphilis testing, diagnosis and treatment or sites for syphilis testing only. These were primary-care centres or community and township hospitals. A total of 363 sites across Yunnan province, including all the 130 antenatal clinics, were officially designated as testing, diagnosis and treatment sites by the provincial authority to ensure that each of 129 districts or counties had at least one such site. An additional 307 testing-only sites were established by equipping existing facilities with treponemal point-of-care test kits (immunochromatographic strips for finger-prick blood).

In 2012, 436 (81.0%) of the 538 sites in place could provide both treponemal testing (*Treponema pallidum* particle agglutination, enzyme immunoassay or immunochromatographic assay) and non-treponemal testing (toluidine red unheated serum test or rapid plasma reagin test). By 2015 the number of sites had increased to 670, of which 635 (94.8%) were equipped with both type of tests. Outreach teams from community-based organizations were also equipped to conduct point-of-care tests. The cost to patients of a syphilis test is normally 20 Chinese yuan or about 3 United States dollars (US$; 6.8 Chinese yuan = 1 US$ in 2017), but the programme provided testing free-of-charge in all the designated antenatal care settings.

To encourage people to seek syphilis screening, health promotion efforts were integrated into the ongoing HIV health education programme, via leaflets, posters and AIDS Day health promotion activities. An opt-out screening for syphilis was offered to people attending sexually transmitted disease clinics, voluntary counselling and testing sites, and methadone maintenance treatment clinics. For people testing positive at the designated testing, diagnosis and treatment sites, treatment was started immediately with intramuscular injection of benzathine benzylpenicillin 2.4 million units (MU) weekly for 2‒3 weeks. For those testing positive at testing-only sites or at outreach services, staff gave patients a referral card with the address, telephone number and contact person at the nearest site where the diagnosis could be confirmed and treatment provided. Pregnant women were routinely screened for syphilis at their first visit to an antenatal clinic and, if positive, treated for infection at the same clinic.

Training and re-training of local staff on how to perform point-of-care testing or on health promotion was a mandate of designated local centres for disease control. Approximately 4000 people annually were trained or re-trained in 1‒2 day training courses at provincial, district and county levels. The trainers were disease control specialists, doctors, nurses and outreach paramedical workers.

To improve implementation of the testing, referral and treatment services, a pay-for-performance scheme was introduced in 2011. The scheme consisted of two mechanisms. The first was a purchase mechanism, by which public health subsidies were provided through an outsourcing contract to compensate sites for the services provided. Specifically, health-care providers at testing-only sites or outreach teams were paid 20 yuan for successful referral of a person positive for syphilis. Providers at testing, diagnosis and treatment sites were paid 20 yuan (US$ 3.03) for treating a referred patient with the recommended regimen. The second mechanism was an assessment by the local health authority of the performance of the sites in delivering these services. Advice and recommendations were provided after the site assessment, and payment was made if performance met the requirements.

## Relevant changes

**Fig. 1:**
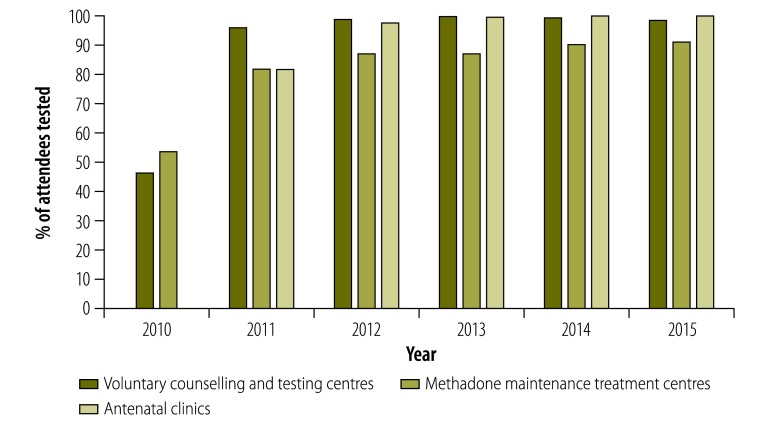
Percentage of people (n= 4 196 543) tested for syphilis when attending different access points in Yunnan province, China, 2010–2015

Notes: Numbers of people attending clinics and confirmed positive for syphilis are shown in Table 1. The National Programme for Prevention and Control of Syphilis in China was launched in 2010. Syphilis screening in antenatal clinics started in 2011 and was part of a separate programme.

The programme’s data collection and reporting system showed that over the period 2011‒2015, a total of 394 643 attendees were tested for syphilis at voluntary counselling and testing centres and 89 497 at methadone maintenance treatment clinics. The proportion of people tested for syphilis increased from 46.2% (32 877 out of 71 162 attendees) in 2010 to 98.2% (68 012/69 259) in 2015 at voluntary counselling and testing centres (Table 1 and Fig. 1). A similar increase was found among drug users attending methadone maintenance treatment clinics, from 53.4% (9836/18 419) in 2010 to 90.8% (17 921/19 737) in 2015. The coverage of testing in antenatal clinics increased from 81.6% (637 241/780 933) in 2011 to 99.7% (730 474/732 893) in 2015.

**Table 1:** Numbers of people tested and treated for syphilis at different access points in Yunnan province, China, 2010–2015

Access point	No. of people					
	2010	2011	2012	2013	2014	2015
**Tested for syphilis**						
Voluntary counselling and testing centres^a^						
Attended clinic	71 162	83 392	85 275	82 511	81 714	69 259
Tested	32 877	79 890	83 911	81 851	80 979	68 012
Methadone maintenance treatment centres^a^						
Attended clinic	18 419	19 945	21 201	21 279	20 406	19 737
Tested	9 836	16 295	18 381	18 555	18 345	17 921
Antenatal clinics^a^						
Attended clinic	N/A	780 933	805 753	759 086	764 008	732 893
Tested	N/A	637 241	786 334	753 696	761 945	730 474
**Treated for syphilis**						
Testing, diagnosis and treatment centres^b^						
Confirmed positive	993	N/A	N/A	1 343	12 976	549
Treated	264	N/A	N/A	643	7 786	453
Antenatal clinics^a^						
Confirmed positive	N/A	239	690	1 032	1 338	1 360
Treated	N/A	164	586	957	1 237	1 320

N/A: data not available.

^a^ Data from routine reporting by clinics.

^b^ Data from ad hoc surveys by the provincial centre for disease control, except those for 2014 which used data from local surveys.

Notes: The National Programme for Prevention and Control of Syphilis in China was launched in 2010. Syphilis screening in antenatal clinics started in 2011 and was part of a separate programme. Syphilis-infected cases were treated with benzathine benzylpenicillin according to the national guidelines.^11^

**Fig. 2:**
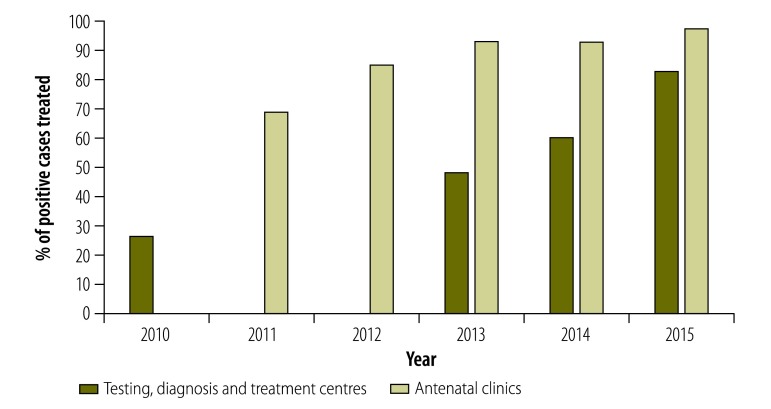
Percentage of people (n= 22 556) treated for syphilis at different access points in Yunnan province, China, 2010–2015

Notes: Numbers of people treated for syphilis are shown in Table 1. The National Programme for Prevention and Control of Syphilis in China was launched in 2010. Data from testing, diagnosis and treatment sites were unavailable for 2011 and 2012. Syphilis screening in antenatal clinics started in 2011 and was part of a separate programme. Syphilis-infected cases were treated with benzathine benzylpenicillin according to the national guidelines.^11^

The percentage of syphilis-infected cases treated with benzathine benzylpenicillin according to the national guidelines^12^ increased from 26.6% (264/993) in 2010 to 82.5% (453/549) in 2015 in testing, diagnosis and treatment sites and from 68.6% (164/239) in 2011 to 97.1% (1320/1360) in 2015 in antenatal clinics (Table 1 and Fig. 2).

Data from sentinel surveillance indicated a decline in syphilis prevalence from 4.2% (252/6000) in 2012 to 3.4% (204/6000) in 2015 among male patients attending sexually transmitted disease clinics and from 2.1% (515/24 500) to 1.2% (269/22 400) among female sex workers.

The mean cost for the additional investment in pay-for-performance was around US$ 39 000 annually (range: US$ 35 000‒70 000).

## Lessons learnt

A specific effort in screening and treatment of syphilis in Yunnan has resulted in an increased uptake of syphilis testing and corresponding increase in treatment of infected cases. In addition to health promotion efforts which have been active for some time, two factors may have contributed to the increases. First, there was a province-wide HIV prevention and control programme in place in which the syphilis intervention services could be easily integrated to increase the uptake of syphilis testing. Second, the pay-for-performance scheme appeared to motivate health-care professionals to undertake syphilis intervention services.

The main lessons learnt are summarized in Box 1. Introduction of point-of-care tests for syphilis served as a facilitator for scaling-up of testing for syphilis,^13^^,^^14^ thereby creating a testing network covering 670 sites across Yunnan province. Furthermore, integrating syphilis testing into the existing services in government health facilities as well as the outreach services of community-based organizations resulted in an increased proportion of people tested and treated for syphilis. One of these strategies, pay-for-performance has been widely implemented in many countries, with varying levels of success.^15^^,^^16^

Box 1 **Summary of main lessons learnt**

- Syphilis screening and treatment services were integrated into the existing human immunodeficiency virus programme as public health interventions in Yunnan province.
- Establishing a network of testing-only and testing, diagnosis and treatment sites across the province facilitated people’s access to syphilis screening and treatment services.
- Introduction and implementation of a pay-for-performance scheme appeared to motivate providers to increase the provision of syphilis testing, referral and treatment services.

Despite the successes, several challenges remain. Although the costs are not high in relation to the provincial government budget for sexually transmitted disease control (more than US$ 2.5 million), the sustainability of local investments in pay-for-performance is a challenge. Treatment for chlamydia or other sexually transmitted infections did not benefit from the pay-for-performance scheme and may have been neglected. We therefore need to determine whether the benefits of introducing specific incentives for syphilis testing outweighed any potential adverse impact on other services. We also need to know whether implementation of pay-for-performance compromises the voluntary nature of testing.

In summary, introduction of financial incentives to providers could be linked to increased uptake of syphilis testing and treatment in the study area. These increases reinforce the need to collect further information on the feasibility, acceptability and cost–effectiveness of introducing pay-for-performance within the national or provincial health services and to translate evidence to policy for responding to the syphilis epidemic in China.
